# Synthesis of 0.75Pb(Zr_0.52_Ti_0.48_)O_3_-0.25BiFeO_3_ Thin Film Capacitors with Excellent Efficiency and Thermal Stability

**DOI:** 10.3390/molecules30010008

**Published:** 2024-12-24

**Authors:** Li Wu, Feifei Han, Kaiyuan Chen, Jianming Deng, Laijun Liu, Biaolin Peng

**Affiliations:** 1 Guangdong Provincial Key Laboratory of Electronic Functional Materials and Devices, Huizhou University, Huizhou 516001, China; winwuli@163.com; 2 Guangxi Key Laboratory of Calcium Carbonate Resources Comprehensive Utilization, College of Materials and Environmental Engineering, Hezhou University, Hezhou 542899, China; cky784813760@foxmail.com; 3 Guangxi Key Laboratory of Optical and Electronic Materials and Devices, Guilin University of Technology, Guilin 541004, China; ljliu2@163.com; 4 School of Advanced Materials and Nanotechnology, Xidian University, Xi’an 710126, China

**Keywords:** PZT, BFO, energy storage, thermal stability

## Abstract

The advancement of miniaturizing electronic information technology draws growing interest in dielectric capacitors due to their high-power density and rapid charge/discharge capabilities. The sol-gel method was utilized to fabricate the 0.75Pb(Zr_0.52_Ti_0.48_)O_3_-0.25BiFeO_3_ (PZT-25BFO) thin film. Excitingly, PZT-25BFO thin film exhibits an exceptional capacitive energy storage density (*W_rec_* = 24.61–39.76 J/cm^3^) and a high efficiency (*η* = 53.78–72.74%). Furthermore, the dielectric energy storage density and efficiency enhance simultaneously with increasing thickness of the thin film. However, the loss factor shows the opposite trend. Specifically, the 12-layer PZT-25BFO thin film demonstrates the optimal properties, boasting a significant energy storage density (15.73 J/cm^3^), a high efficiency (77.65%), and remarkable thermal stability (±0.55% variation) from 303 K to 383 K at 1000 kV/cm. This excellent thermal stability can be attributed to the residual stress resulting from a phase transition from the rhombohedral to tetragonal phase. The result offers valuable guidance for the development of ferroelectric thin films in high-power capacitive energy storage applications.

## 1. Introduction

Dielectric capacitors with exceptional power density and rapid charge/discharge capabilities have exhibited great potential for electronic device applications in recent years [1,2]. Dielectric/ferroelectric materials and devices have attracted considerable attention because of their notable characteristics such as high spontaneous polarization (*P*_max_), thermal stability, and mechanical stability [3,4]. Currently, researchers are focused mainly on investigating the capacitive energy storage capabilities of bulk ferroelectric ceramics [5,6,7]. In contrast to the electric properties of thin film materials, bulk ferroelectric materials exhibit a comparatively low breakdown strength (*BDS*) and capacitive energy storage density. Therefore, thin film materials with a large difference between remnant polarization (*P*_r_) and *P*_max_ are anticipated to exhibit exceptional capacitive energy storage capabilities and high efficiency even at a low-drive voltage.

Pb(Zr*_x_*Ti_1−*x*_)O_3_ (PZT) has gained attention in the field of ferroelectric film materials due to its exceptional electrical properties, such as dielectric relaxor behavior, excellent piezoelectric coefficient, ferroelectric polarization [8,9,10,11], and high-power capacitive energy at the morphotropic phase boundary (MPB) [12,13]. However, PZT thin films incorporate Pb ions and easily generate oxygen vacancies at high temperatures, resulting in a decline in ferroelectric polarization and an increase in the coercive field [14,15]. Chemical modification through the occupation of various cations is a highly successful method for enhancing the electrical characteristics of solid solutions. PZT thin films frequently modify the various behaviors at the A-site and/or B-site in ABO_3_ perovskite through the addition of dopants, such as BiFeO_3_ [16], La^3+^ [17,18], Sn^2+^ [19], Nb^5+^ [20], etc. It is noted that BiFeO_3_ is a lead-free multiferroic compound that possesses Bi^3+^ ions with lone pair electrons, as well as Fe^2+^ and Fe^3+^ ions with complex valence electrons. It has been found to exhibit the highest theoretical spontaneous polarization (90 μC/cm^2^ < *P*_s_ < 100 μC/cm^2^) so far [21,22]. The PZT with a Zr/Ti ratio of 52/48, close to the MPB, exhibits exceptional electrical characteristics at room temperature [23,24,25]. Hence, it is essential to investigate the PZT with composition near MPB doped BFO system. Lappalainen et al. found a correlation between elevated residual stress and decreased dielectric properties [26]. Tuttle et al. [27] proposed that residual stress has a considerable impact on the preferred orientation of domains. The results demonstrate that controlling residual stress during the preparation of the film could enhance the performance of capacitive energy storage.

To investigate the capacitive energy storage characteristics and establish the correlation between residual stress and capacitive energy storage performance, a sol-gel process was employed to fabricate 0.75Pb(Zr_0.52_Ti_0.48_)O_3_-0.25BiFeO_3_ (PZT-25BFO) thin films. The ratio of Zr/Ti of the thin film was exquisitely designed to 0.52/0.48 [8], as shown in Figure S1a, where the rhombohedral ferroelectric and tetragonal ferroelectric phases coexist. Excitingly, PZT-25BFO thin film exhibits an exceptional capacitive energy storage density (*W_rec_* = 24.61–39.76 J/cm^3^) and a high efficiency (*η* = 53.78–72.74%). The loss factor showed an inverse relationship with the capacitive energy storage density and efficiency as the thickness of PZT-25BFO film increased. The 12-layer PZT-25BFO thin film achieved a high capacitive energy storage density of 15.73 J/cm^3^ and high efficiencies of 77.65% at 1000 kV/cm. Additionally, the thin film exhibited super-high thermal stability (±0.55% variation) from 303 K to 383 K. This remarkable performance is ascribed to residual stress originating from a phase transition from the rhombohedral to the tetragonal phase. The result provides a guideline for the development of high-power capacitive energy storage applications.

## 2. Results and Discussion

### 2.1. Structure

**Figure 1:**
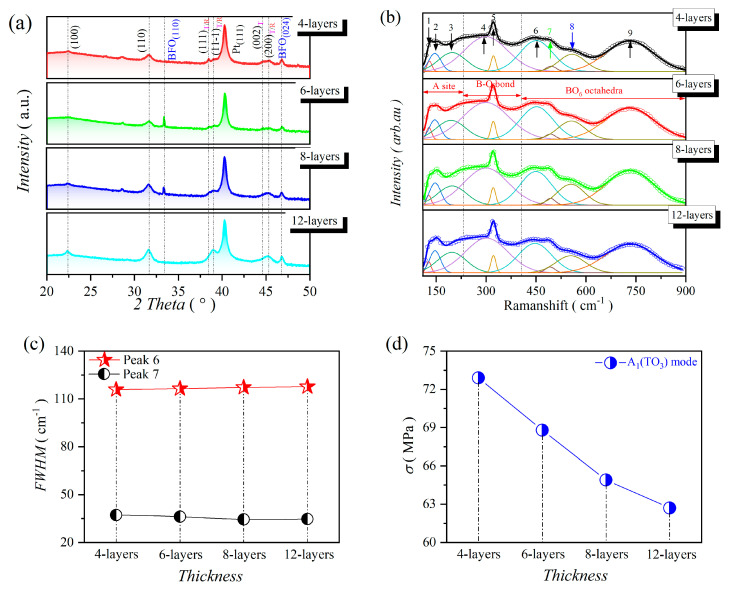
Phase structure of 4, 6, 8 and 12-layer PZT-25BFO thin films (**a**) XRD patterns, (**b**) the Raman scattering spectra fitting to nine Gaussian–Lorentz peak functions, (**c**) Raman intensity and FWHW variation with respect to peaks 6 and 7, (**d**) The Raman vibration mode A_1_(TO_3_) with peak 8.

PZT-25BFO thin films consisting of four, six, eight, and 12 layers were deposited onto Pt(111)/TiO*_x_*/SiO_2_/Si(100) substrates, respectively. The XRD patterns of PZT-25BFO thin films with varying layers were examined and presented in Figure 1a. All thin film samples displayed excellent crystallinity and almost pure perovskite phase with a strong (111) preferential orientation, while weak peaks of (100) and (110) are also observed. A tiny number of secondary phases (bismuth ferrite) can be detected due to the excess of Bi (see (110/024)) [16]. In addition to the rhombohedral ferroelectric (*R*_FE_) phase (see (111/11-1), the tetragonal ferroelectric (*T*_FE_) phase (see (002/200), etc.) can also be detected [28]. As the number of film layers increased, the two reflection peaks (111)_T/R_ of rhombohedral phase and (11-1)_T/R_ of tetragonal phase combined into the (111)_T/R_ one, and the preferred orientation changes from (111) to (100). It is noted that the (002)_T_ and (200)_T/R_ peaks can be detected on the 4-layer thin film, and the intensity becomes more and more strong with increasing the number of film layers. Due to the composition of carefully designed PZT thin film, which is close to the MPB, it allows the coexistence of *R*_FE_ and *T*_FE_ phases [25].

The Raman scattering spectra of PZT-25BFO thin films were acquired to obtain additional insights into the local phase structure and the proportions of *T*_FE_ and *R*_FE_ phases, as illustrated in Figure 1b. Nine distinct Gaussian–Lorentz peaks were utilized to fit the Raman spectra. Raman peaks 1 (128 cm^−1^) and 2 (145 cm^−1^) corresponded to the E_1_(TO_1_) and A_1_(TO_1_) modes, respectively. These are associated with the vibrations of A-site ions in perovskite structure [29,30,31,32]. The appearance of the Raman peak 2 indicates the existence of *R*_FE_ phase in PZT-25BFO thin film. The Raman peaks 3, 4, and 5, appeared around 198, 300, and 321 cm^−1^ corresponding to A_1_(TO_1_), E(TO_4_), and A_1_(TO_2_) modes, respectively [33,34]. The appearance of peak 5 is owing to the asymmetric (Fe/Zr/Ti)-O phonon vibrations in the *T*_FE_ phase [31,32]. The A_1_(LO_2_) and E(TO_3_) modes around 450 and 494 cm^−1^ designated as peak 6 and 7, respectively. These are associated with the symmetric stretching of O-Fe/Ti/Zr-O in tetragonally distorted polar [(Ti/Zr/Fe)O_6_] octahedral clusters [35,36,37]. Peak 8 is the A_1_(TO_3_) mode (556 cm^−1^) caused principally by residual stress. It is shifted slightly to a lower frequency as the film thickness increases [32,38,39,40]. Peak 9 (706 cm^−1^) is the E(LO_3_) mode, which is distributed to the (Zr/Ti)-O torsional modes [41]. The full width at half maximum (FWHM) values of the selected Raman peaks of PZT-25BFO are presented in Figure 1c. The FWHM of peak 6 showed slight variation as the number of film layers increased. Moreover, the FWHM of peak 7 initially decreased and then reached a steady plateau as the number of film layers increased. It indicates that the proportion of *T*_FE_ phase reduced and showed no significant change as the number of film layers increased. The result is consistent with the XRD analysis. As depicted in Figure 1c, the A_1_(TO_3_) mode of PZT-25BFO thin film shifted slightly to low frequency as the number of film layers increased. The change in Raman shift is probably associated with the grain size and the residual stress [42,43,44]. However, the SEM micrographs of PZT-25BFO thin film do not show any noticeable differences in grain size (see the following section). Therefore, the Raman shift of the A_1_(TO_3_) mode originated from the residual stress of PZT-25BFO thin film. To comprehend the relationship between film layers and residual stress, the Lydane–Sach–Teller function was defined as follows [38]:

(1) $$\sigma =\sigma_{0}\left[1-{\left(\omega /\omega_{0}\right)}^{2}\right]$$

where *σ*_0_ represents the stress level when the phonon frequency is zero, *ω*_0_ denotes the phonon frequency when there is no stress. According to Xu et al. [32], the Lydane–Sach–Teller function relationship was utilized to determine the Raman shift of the A_1_(TO_3_) mode (peak 8) for PZT thin films, with *σ*_0_ = 640.7 MPa and *ω*_0_ = 528.8 cm^−1^. In PZT-25BFO thin film with 4, 6, 8, and 12-layers, the Raman shift of the A_1_(TO_3_) modes was observed at 558.14, 556.48, 554.96, and 554.07 cm^−1^, respectively. Based on the Lydane–Sach–Teller function, the residual stresses of thin films with 4, 6, 8 and 12-layer were 72.9, 68.8, 64.9 and 62.7 MPa, respectively, as shown in Figure 1d. The residual stress and film layers showed the opposite trend in PZT-25BFO thin film. The thin film had a relatively low residual stress as the number of film layers increased, due to a reduction in the clamping effect of the substrate [45]. A phase transition from rhombohedral to tetragonal phase is observed by XRD and Raman spectroscopy.

**Figure 2:**
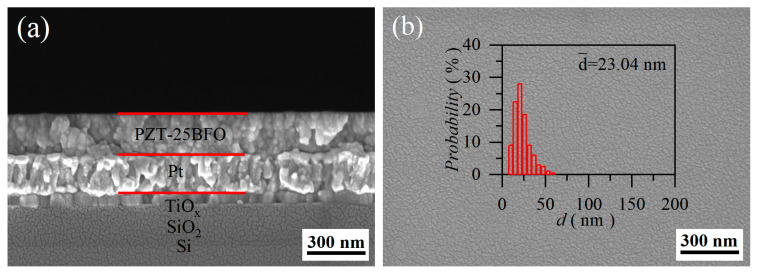

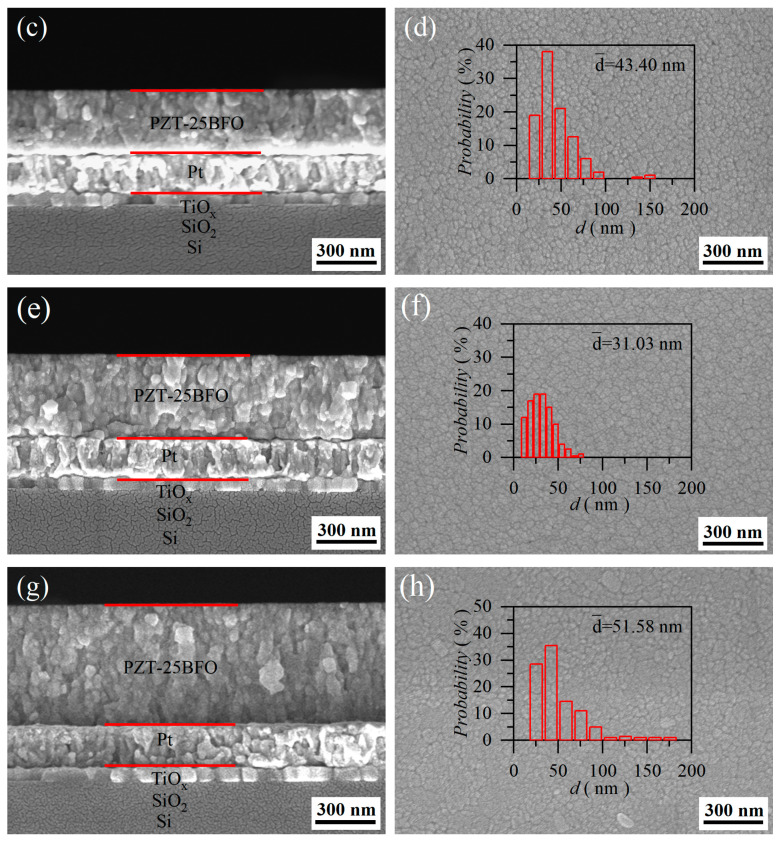
The cross-sectional SEM images of PZT-25BFO thin films (**a**) 4, (**c**) 6, (**e**) 8 and (**g**) 12-layer. The surface SEM images of PZT-25BFO thin films (**b**) 4, (**d**) 6, (**f**) 8 and (**h**) 12-layers (Insets: statistic distribution diagram of grain sizes).

The SEM images in Figure 2a,c,e,g illustrate the cross-sectional morphologies of PZT-25BFO thin film. Figure 2b,d,f,h display the surface images of thin films with 4, 6, 8 and 12 layers, respectively. The grain sizes of the thin films with 4, 6, 8 and 12-layers were approximately 225, 350, 425, and 600 nm, respectively. The thin films demonstrated a uniform surface microstructure devoid of any cracks. The grain size slightly increased as the number of thin film layers increased. The average grain sizes of PZT-25BFO films were 23.04, 43.40, 31.03 and 51.58 nm for 4 to 12-layer, respectively. According to Tunkasiri’s work, the effect of grain size on *BDS* could be described by *E* ∞ *G*^-a^, where *E*, *G*, and *a* are the *BDS*, grain size, and a constant, respectively [46]. Thus, it is expected that the small grain size of PZT-25BFO films can improve the *BDS*. Moreover, the result of chemical analysis by energy dispersive spectroscopy (*EDS*) in cross-sectional SEM revealed that the composition of the thin film is consistent with its chemical formula, as shown in the inset of Figure S2.

**Figure 3:**
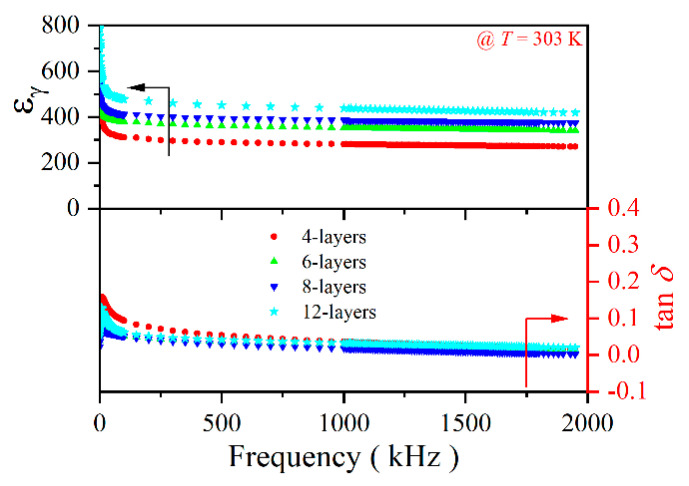
Frequency dependence of *ε_γ_* and tan *δ* of the 4, 6, 8 and 12-layer PZT-25BFO thin films at 303 K.

Figure 3 displays the relationship between frequency and dielectric permittivity (*ε_γ_*) and loss factor (tan *δ*) of the 4, 6, 8 and 12-layer PZT-25BFO thin films. The *ε_γ_* increased proportionally as the number of film layers increased, and the value of *ε_γ_* with the PZT-25BFO film remains stable over a broad frequency range, for which the value is about ~400. The 12-layer PZT-25BFO thin film possesses the maximum *ε_γ_* among all the samples. The *ε_γ_* of thin films is influenced by various parameters related to the electrical properties, including grain size, orientation, and residual stress [44]. According to the SEM images, the grain size of PZT-25BFO thin films slightly increased as the number of layers increased. This suggests that the influences of grain size on dielectric properties of PZT thin films can almost be neglected. Hence, the primary determinants of *ε_γ_* are orientation and residual stress. First, PZT-25BFO thin films were orientated in the (111) direction. The 12-layer PZT-25BFO thin film possesses the maximum *ε_γ_*, which can be attributed to its high proportion of the tetragonal phase. Second, the residual stress of the 12-layer PZT-25BFO thin film is smallest. It corresponds well with the Raman result.

### 2.2. Energy Storage Density

**Figure 4:**
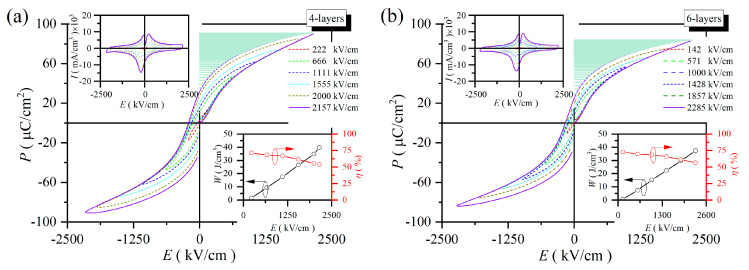

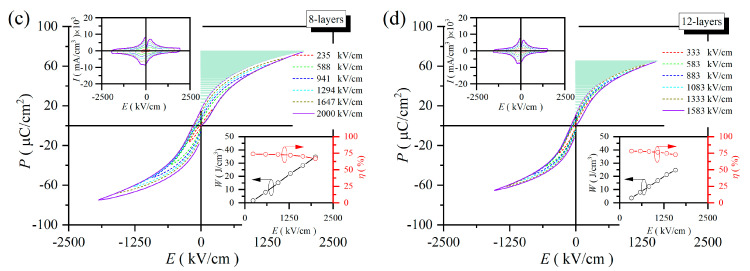
The *P-E* loops of PZT-25BFO thin films at the selected electric field (**a**) 4, (**b**) 6, (**c**) 8 and (**d**) 12-layer [Insets: *I* (*E*), the *W* (*E*) and *η* (*E*)].

Figure 4 depicts the *P-E* loops of 4, 6, 8 and 12-layer PZT-25BFO thin films at various electric fields at room temperature. All *P*-*E* loops showed the characteristic of relaxor ferroelectric with negligible leakage current under the driving field. The variation of the *P*_max_ value of all the samples followed a similar trend to the dielectric permittivity. The *P-E* loops of 12-layer thin film are narrower compared to other thin films. This phenomenon can be explained by a reduction in the concentration of space charges and oxygen vacancies in thin films [47]. As the number of film layers increased, however, tensile stress decreased and crystalline quality improved, which is further verified by Raman. The values of *W* and *η* were determined within the range from 142 kV/cm to the critical *BDS*, as illustrated in the insets of Figure 4. The *W* value showed a slight increasing trend as the applied electric field increased. When *BDS* was below 2157 kV/cm, the 4-layer thin film displayed the highest *W* (39.76 J/cm^3^) and the lowest *η* (53.78%). The *P*_max_ − *P*_r_ values are 59.15, 59.14, 59.41, and 54.76 μC/cm^2^ for 4, 6, 8 and 12-layer films at 2157, 2285, 2000 and 1583 kV/cm, respectively. It can be found that the 4-layer film possesses highest *W*, attributed to the high *P*_max_ − *P*_r_ and the high electric field. Furthermore, the 12-layer thin film exhibited the highest *η* (72.74%) at 1584 kV/cm, which can be attributed to its comparatively low *P*_r_ value in comparison to the other samples. Due to the testing limitations of the instruments, the 12-layer thin film did not achieve its maximum *BDS*. The 12-layer thin film yielded the highest *W* value of 24.61 J/cm^3^. The comparison of *W*_max_, *η*, and *BDS* in this work with that of other thin films, including 0.7NBT-0.3ST [48], 0.8PMN-0.2PT [49], BNT-ST [50], PLZST [51] and 0.4BNZ-0.6PT [52], are shown in Table 1.

Low leakage current and high *BDS* are the advantage of miniaturization and integration of electronic components in the large discharge capacitive *W*. Figure S3a illustrates the current leakage curves at room temperature for various layers of PZT-25BFO thin films at 250 kV/cm. There were no breakdowns in any of the samples during the transients that lasted more than 1000 ms, even throughout repetitive testing. The leakage current for films with 4, 6, 8 and 12-layers were 0.25, 4.00, 0.95 and 0.17 nA, respectively. The *BDS* is crucial in verifying the effectiveness of the electric field and the density of energy storage. To gain a deeper comprehension of the applications in capacitive energy storage, the *BDS* of PZT-25BFO films was examined using the Weibull distribution [48,53], as illustrated in Figure S3b. According to the Weibull distribution, the average *BDS* can be calculated by intersecting the linear fitting line with the horizontal axis. The *BDS* values of the PZT-25BFO films for 4, 6, 8 and 12-layers were 2043, 2001, 2062 and 1975 kV/cm, respectively. Clearly, the *BDS* of all thin is films much higher than 1000 kV/cm (the safety electric field applied). Moreover, the decrease of grain size and increase of density are beneficial to the high *BDS* [54]. Thus, all thin films with high *BDS* are related to the small grain size. This result has a positive effect on the improvement of energy storage efficiency.

**Figure 5:**
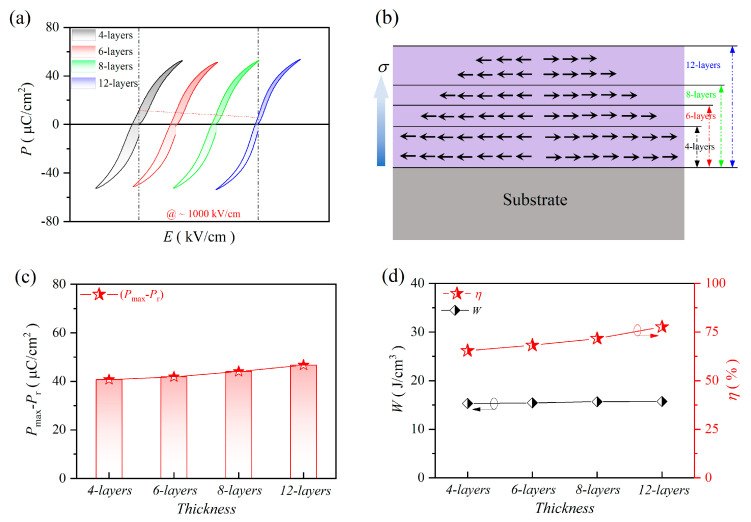
The ferroelectric characteristics of 4, 6, 8 and 12-layer PZT-25BFO films (**a**) *P-E* loops subjected to an electric field of approximately 1000 kV/cm, (**b**) Schematic diagrams of in-plane thermally induced residual stress (*σ*) in thin films. (**c**) *P*_max_ − *P*_r_ values. (**d**) The *W* (*E*) and *η* (*E*) at room temperature.

To assess the potential applications of PZT-25BFO thin films in energy storage devices, the thermal stability was investigated, as shown in Figure 5. The *P*-*E* loops of PZT-25BFO thin film measured at room temperature and 1000 kV/cm in Figure 5a. With the increase of film thickness, there is no significant variation in *P*_max_, which stabilizes at a value of 52.60 μC/cm^2^. As illustrated in Figure 5b, c, the *P*_r_ value decreased and the *P-E* loops became progressively narrower as the number of film layers increased, which corresponded to the residual stress. Table 2 shows the *P*_max_ − *P*_r_, *P*_r_, *W*, and *η* values for 4, 6, 8 and 12-layers PZT-25BFO films. As the film thickness increased, the *W* value approached a constant, whereas the *η* value gradually increased. The *η* value is directly proportional to the *W*/(*W*_loss_ + *W*) ~ *η*. The 12-layer thin film exhibited the lowest *W*_loss_ and the highest *W*, as depicted in Figure 5d. The *η* value of the 12-layer thin film is the highest. Thus, the 12-layer thin film has exceptional performance in capacitive energy storage.

**Figure 6:**
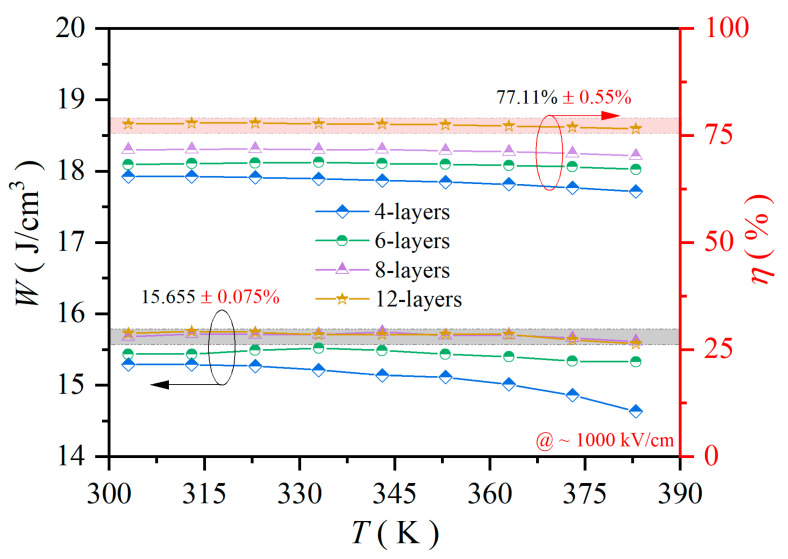
The *W* (*E*) and *η* (*E*) values of PZT-25BFO thin films with 4, 6, 8 and 12-layers, respectively, at temperatures ranging from 303 K to 308 K.

Thermal stability is an essential characteristic for the practical applications of capacitors. Figure 6 displays the relationship between temperature and the calculated values of *W* and *η* of PZT-25BFO thin films. These *P-E* loops were monitored ranging from 303 K to 383 K at 1000 kV/cm (Figure S4). The *W* first increased and then remained nearly constant. The maximum *W* was around 15.73 J/cm^3^. The change in *W* from 303 K to 383 K was approximately 0.15%. The thermal stability of PZT-25BFO thin film was significantly superior to that of polymer [55] and HfO_2_-based materials [56]. This evidence demonstrates that the thin film is a viable option for dielectric capacitors operating in harsh environments. The thermal stability of the *η* increased as the layer thickness of PZT-25BFO film increased. The *η* value of the 12-layer film remained nearly constant at approximately 77.65% across the whole temperature range. The change in *η* from 303 K to 383 K was less than ± 0.55%. Therefore, the excellent thermal stability can be attributed to the correlation among residual stress, gain size, and the phase proportion.

**Figure 7:**
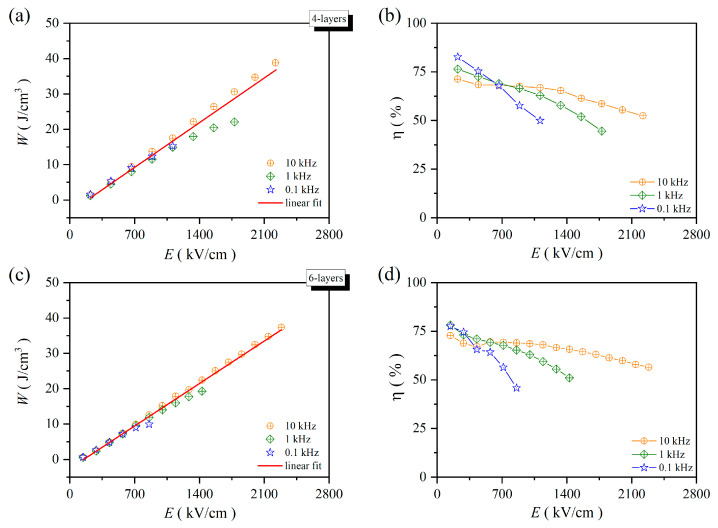

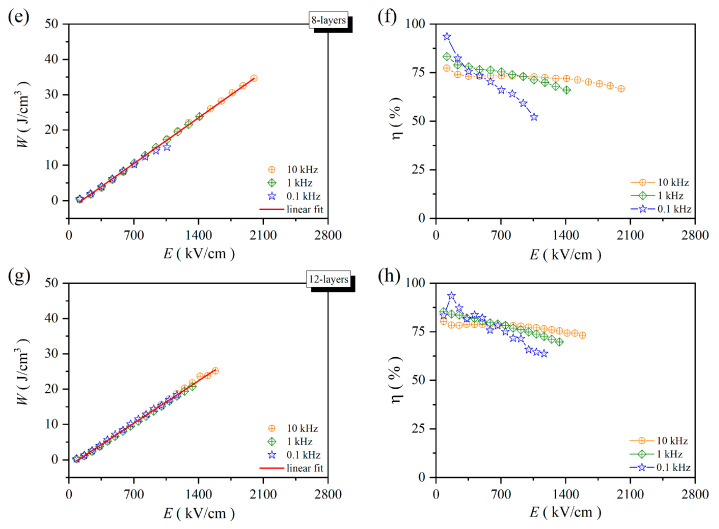
The *W* of PZT-25BFO thin films vs. the driver electric fields of 100 Hz, 1 kHz and 10 kHz at room temperature (**a**) 4, (**c**) 6, (**e**) 8 and (**g**) 12-layer. The *η* of PZT-25BFO thin films vs. the driver electric fields of 100 Hz, 1 kHz and 10 kHz at room temperature (**b**) 4, (**d**) 6, (**f**) 8 and (**h**) 12-layer.

Figure 7 illustrates the calculated capacitive energy storage density and efficiency of PZT-25BFO film. The frequency dependence of capacitive energy storage decreased as the number of thin film layers increased. In capacitive energy storage, the relationship between frequencies and electric fields follows a linear function. At 1 kHz, the *η* value of the 12-layer thin film exhibited a fluctuating range from 69.69% at 1333 kV/cm to 85.37% at 83 kV/cm. Therefore, the 12-layer PZT-25BFO thin film demonstrates superior frequency-dependent energy storage stability.

## 3. Characterization

The crystal structure of PZT-25BFO thin films was determined using X-ray diffraction (XRD, MRD DY05569, Tokyo, Japan). The surface and cross-section of the films were analyzed using scanning electron microscope (SEM, ZEISS, sigma 500, Berlin, Germany). Raman scattering spectra were acquired using a Labram HR Evo Raman spectrometer. To evaluate the electrical performance, 90 μm-long Au/Cr square dots were evaporated using RF magnetron sputtering with a shadow mask. The ferroelectric properties were measured using a ferroelectric tester (Precision Premier II Radiant Technologies Inc, Washington, USA). The temperature was closely monitored using a highly accurate thermal controller (Linkam, THMSG600, London, UK). The dielectric performance was determined utilizing a precision impedance analyzer (Agilent, E4980A, Hefei, China). To obtain the average dielectric breakdown strength (*BDS*), 22 points were randomly selected for each PZT-25BFO sample. The recoverable energy density (*W*) and efficiency (*η*) were calculated utilizing Equations (2) and (3), respectively [57,58,59,60].

(2) $$W=\int_{P_{r}}^{P_{\max}}E\;dP$$

(3) $$\eta =\frac{W}{W+W_{\mathrm{loss}}}$$

where *P*_max_ represents the maximum polarization, *P*_r_ denotes the remnant polarization, and *P* is the polarization value at the applied field *E*. The energy loss density, denoted as *W*_loss_, is obtained by integrating the area of the closed *P-E* hysteresis loop. Large *P*_max_, small *P*_r_, high *E*, and low *W*_loss_ are crucial for achieving high *W* and *η* in dielectric capacitors. Based on the above Equation (2), in order to get higher energy storage performance, improving the difference between *P*_max_ and *P*_r_ and enhancing *BDS* are the two effective ways [61,62]. This indicates that relaxor ferroelectrics with slim *P*-*E* loops are promising candidates.

## 4. Materials and Methods

### Preparation of PZT-25BFO Thin Films

PZT-25BFO thin films were synthesized using the sol-gel method, as illustrated in Figure S1b. The compound C_6_H_9_BiO_6_ (99.9%, Shanghai Jinpan Biotechnology Co. Ltd., Shanghai, China), with an excess of 20% Bi, and Pb(CH_3_COO)_2_·3H_2_O (99.5%, Sinopharm Chemical Reagent Co. Ltd., Shanghai, China), with an excess of 10% Pb, were dispersed in glacial acetic acid. An additional 1% mol of Mn(CH_3_COO_2_) (99%, Shanghai Jinpan Biotechnology Co. Ltd., Shanghai, China) was added to the aforementioned solution as an additive to reduce leakage current. The compounds C_12_H_28_O4Zr (70 wt.% in propanol, Aldrich), C_16_H_36_O_4_Ti (97%, Shanghai Jinpan Biotechnology Co. Ltd., Shanghai, China), and Fe(C_5_H_7_O_2_)_3_ (99.5%, Shanghai Jinpan Biotechnology Co. Ltd., Shanghai, China) were dissolved in a solution containing a mixture of acetylacetone and glacial acetic acid. The Pb/Bi/Mn solution was added to Zr/Ti/Fe solution, and the resulting mixture was stirred at 80 °C for 1 h. After that, the suitable additives were added to PZT-25BFO solution, as depicted in Figure S1b. The final concentration of PZT-25BFO precursor solution was 0.3 M. Following a 24 h aging period, PZT-25BFO solution was applied onto Pt(111)/TiO*_x_*/SiO_2_/Si(100) substrates, which had been previously washed with acetone and ethanol. PZT-25BFO thin films were deposited using a layer-by-layer approach involving drying, pyrolyzing, and annealing processes, as illustrated in Figure S1b. Spin-coating was performed at 4000 rpm for 40 s on each layer of PZT-25BFO thin film. Each wet layer must be dried at 200 °C for 5 min on a hotplate, pyrolyzed at 400 °C for 5 min, and annealed in a pot at 750 °C for 3 min on a tube furnace, in that order, to prevent the formation of cracks. Finally, the aforementioned steps were repeated multiple times until the desired thickness was achieved.

## 5. Conclusions

The PZT-25BFO thin films were synthesized using a sol-gel technique, resulting in a significant capacitive energy storage density ranging from 24.61 to 39.76 J/cm^3^ and a high efficiency ranging from 53.78 to 72.74%. Based on XRD and Raman analysis, The thin films showed excellent crystallinity and a pure perovskite phase located near MPB where the rhombohedral and tetragonal phases coexist. The tryptic relaxor ferroelectric *P-E* loops demonstrate that the loss factor decreased as the number of layers of thin film increased. Conversely, the capacitive energy storage density and efficiency exhibited the opposite trend with increasing electric field. The 12-layer thin film exhibited a large capacitor energy storage density of 15.73 J/cm^3^, a high efficiency of 77.65% and excellent thermal stability (±0.55% variation) within the temperature range from 303 K to 383 K. The remarkable efficiency and high capacitive energy storage density of PZT-25BFO thin films are ascribed to residual stress originating from a phase transition from the rhombohedral to tetragonal phase. The result offers a guideline for the development of ferroelectric thin film materials used in high-power capacitive energy storage applications.

## Figures and Tables

**Table 1:** Comparison of *W*_max_, *η*, and *BDS* for PZT-25BFO thin films with capacitive energy storage capability.

Materials	*W*_max_ (J/cm^3^)	*η* (%)	*E* (kV/cm)	References
4-layers	39.76	53.78	2157	This work
6-layers	37.37	56.47	2285	This work
8-layers	34.67	66.67	2000	This work
12-layers	24.61	72.74	1584	This work
0.8PMN-0.2PT	31	64	2000	[49]
BNT-ST	36.1	40.8	1965	[50]
PLZST	13.5	73	900	[51]
0.7NBT-0.3ST	27	45.2	1903	[48]
0.4BNZ-0.6PT	39.8	56.5	2167	[52]

**Table 2:** The parameters of *P*_max_ − *P*_r_, *P*_r_, *W*, and *η* for the 4, 6, 8 and 12-layer PZT-25BFO thin films.

Samples	*P*_max_ − *P*_r_ (μC/cm^2^)	*P*_r_ (μC/cm^2^)	*W* (J/cm^3^)	*η* (%)
4-layers	40.71	11.89	15.29	65.37
6-layers	41.83	9.50	15.43	68.20
8-layers	44.07	8.45	15.68	71.64
12-layers	46.69	7.10	15.73	77.65

## Data Availability

The original contributions presented in this study are included in the article/Supplementary Material. Further inquiries can be directed to the corresponding authors.

## Supplementary Materials

The following supporting information can be downloaded at: https://www.mdpi.com/article/10.3390/molecules30010008/s1, Figure S1: (a) The phase diagram of Pb(Zr_x_Ti_1−x_)O_3_ system [8], (b) A flow chart depicting the sol-gel and spin coating processes for PZT-25BFO thin films; Figure S2. Cross-sectional EDS diagram in SEM sample. Inset lists: the atomic percentages of the elements; Figure S3. The ferroelectric characteristics of the PZT-25BFO thin films. (a) Leakage current curves I (t) about different layers’ thin films under ~ 250 kV/cm, (b) The Weibull distribution of the BDS; Figure S4. The P-E loops of PZT-25BFO thin films at temperatures between 303 K and 383 K when subjected to an electric field of around 1000 kV/cm. (a) 4, (b) 6, (c) 8 and (d) 12-layer.
