# Supplementary material for: Synthesis of 0.75Pb(Zr0.52Ti0.48)O3-0.25BiFeO3 Thin Film Capacitors with Excellent Efficiency and Thermal Stability
Source: Molecules. 2024 Dec 24;30(1):8. doi: 10.3390/molecules30010008 (PMC11721792; doi:10.3390/molecules30010008)
Supplement: Supplementary file 1 [file molecules-30-00008-s001.zip › molecules-3358636-supplementary.pdf]

Supplementary Materials for
Synthesis of $0.75Pb(Zr0_{.52}Ti_{0.48})O_3$-$0.25BiFeO_3$ Thin Film Capacitors with Excellent Efficiency and Thermal Stability

Li Wu [1,2], Feifei Han [2,*], Kaiyuan Chen [2], Jianming Deng [1,*], Laijun Liu [3], Biaolin Peng [4,*]

[1]*Guangdong Provincial Key Laboratory of Electronic Functional Materials and Devices, Huizhou University, Huizhou 516001, China*

[2]*Guangxi Key Laboratory of Calcium Carbonate Resources Comprehensive Utilization, College of Materials and Environmental Engineering, Hezhou University, Hezhou 542899, China*

[3]*Guangxi Key Laboratory of Optical and Electronic Materials and Devices, Guilin University of Technology, Guilin 541004, China*

[4]*School of Advanced Materials and Nanotechnology, Xidian University, Xi'an 710126, China*

[*]Correspondence: feifeihanldx@163.com; jmdeng@hzu.edu.cn; pengbl8@126.com

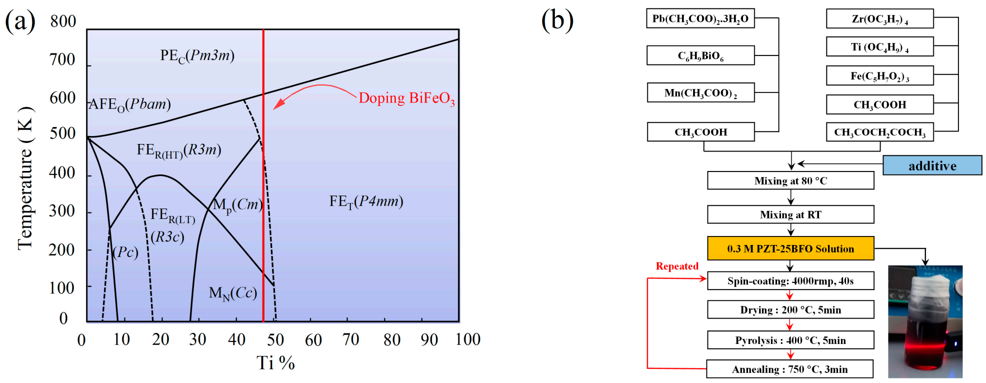

Figure S1. (**a**) The phase diagram of Pb(Zr$_x$Ti$_{1-x}$)O$_3$ system [8], (**b**) A flow chart depicting the sol-gel and spin coating processes for PZT-25BFO thin films.

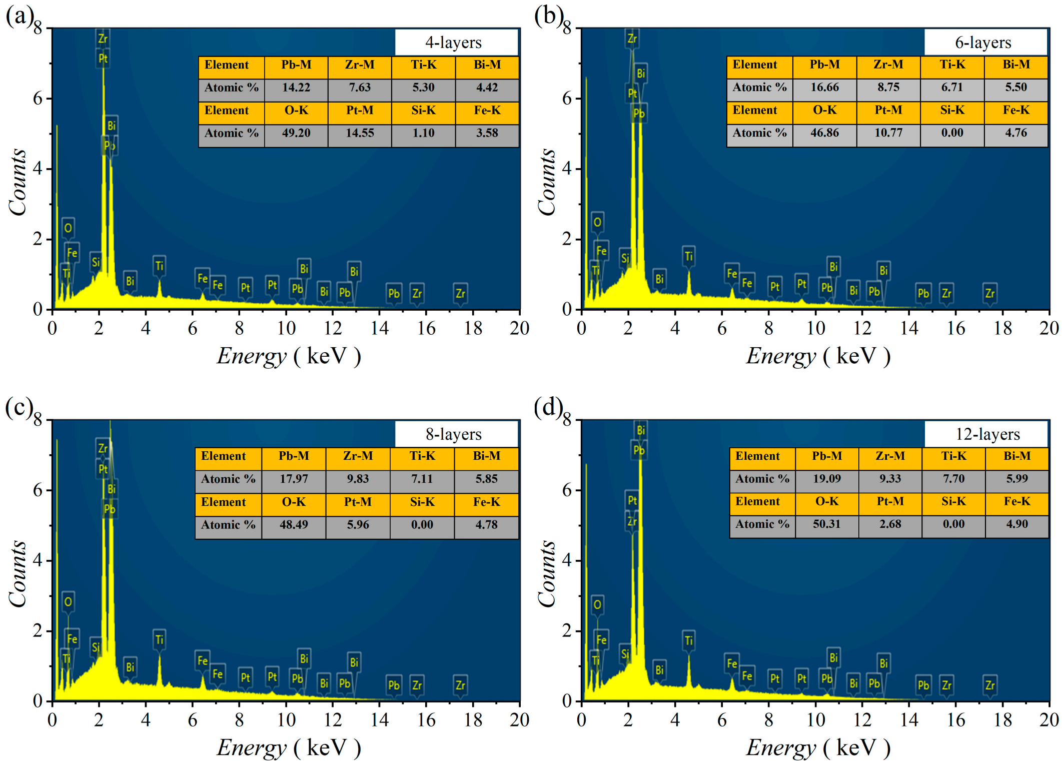

Figure S2. Cross-sectional EDS diagram in SEM sample. Inset lists: the atomic percentages of the elements. (**a**) 4, (**b**) 6, (**c**) 8 and (**d**) 12-layer.

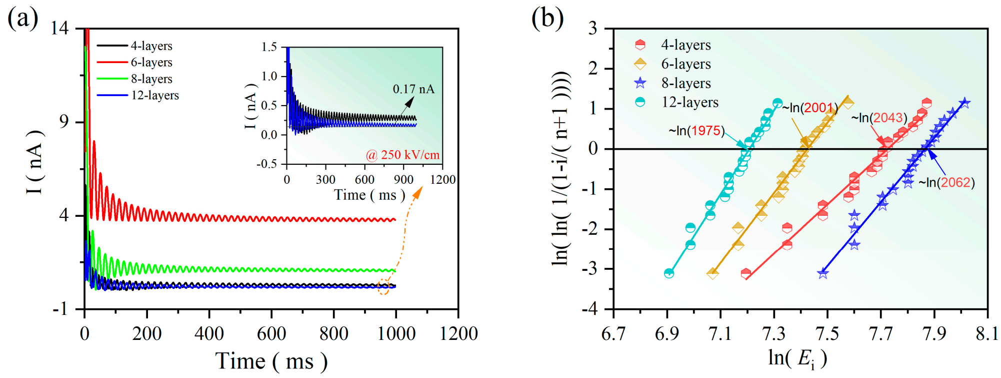

Figure S3. The ferroelectric characteristics of the PZT-25BFO thin films. (**a**) Leakage current curves *I* (t) about different layers' thin films under ~ 250 kV/cm, (**b**) The Weibull distribution of the *BDS*.

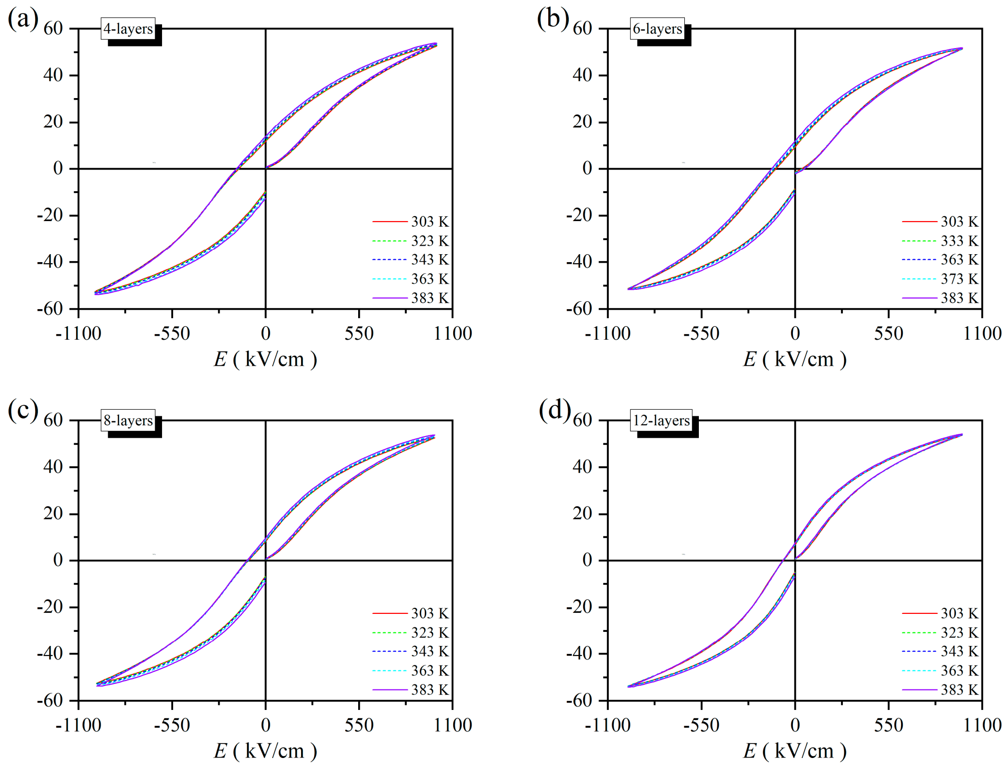

Figure S4. The *P-E* loops of PZT-25BFO thin films at temperatures between 303 K and 383 K when subjected to an electric field of around 1000 kV/cm. (**a**) 4, (**b**) 6, (**c**) 8 and (**d**) 12-layer.
